# Correction to “A New Case of Sodium‐Dependent Multivitamin Transporter Defect Occurring as a Life‐Threatening Condition Responsive to Early Vitamin Supplementation and Literature Review”

**DOI:** 10.1002/mgg3.70105

**Published:** 2025-05-15

Van Vyve F.‐X*, Mercier N*, Papadopoulos J, Heijmans C, Dessy H, Monestier O, Dewulf J, Roland D. “A new case of sodium‐dependent multivitamin transporter defect occurring as a life‐threatening condition responsive to early vitamin supplementation and literature review.” *Molecular Genetics & Genomic Medicine*, 12, (2024): e2388, https://doi.org/10.1002/mgg3.2388.

* F.‐X. Van Vyve and N. Mercier working as co‐first authors. The authors N. Mercier^2^ , C. Heijmans^2^ , H. Dessy^2^ and J.P.Dewulf^4^ are affiliated only with the below mentioned affiliation.

^2.^Department of Pediatrics, CHU HELORA, La Louvière, Belgium.

Therefore, the order of other affiliations is changed accordingly.

F.‐X. Van Vyve^1^, N. Mercier^2^, J. Papadopoulos ^1^, C. Heijmans^2^, H. Dessy², O. Monestier ^3^, J. P. Dewulf^4^, D. Roland^5^

**Affiliations**

- ^1^Department of Pediatrics Intensive Care Unit, CHU Helora, La Louvière, Belgium.
- ² Department of Pediatrics , CHU Helora, La Louvière, Belgium.
- ^3^ Department of Molecular Biology, Institute of Pathology and Genetics, Charleroi, Belgium.
- ^4^Biochemical Genetics and Newborn Screening Laboratory, Department of Clinical Chemistry, Cliniques Universitaires Saint‐Luc, Brussels, Belgium.
- ^5^Department of Human Genetics, Center for Inherited Metabolic Disorders, Institute of Pathology and Genetics, Charleroi, Belgium.

We apologize for this error.

